# Effect of Ab interno XEN gel stent implantation on corneal astigmatism

**DOI:** 10.1371/journal.pone.0338890

**Published:** 2025-12-17

**Authors:** Pawasoot Supasai, Kanwasee Kanjana, Kannawee Boonchuenchom, Yosanan Yospaiboon

**Affiliations:** 1 KKU Glaucoma Center of Excellence, Department of Ophthalmology, Faculty of Medicine, Khon Kaen University, Khon Kaen, Thailand; 2 KKU Eye Center, Department of Ophthalmology, Faculty of Medicine, Khon Kaen University, Khon Kaen, Thailand; Seirei Hamamatsu General Hospital, JAPAN

## Abstract

**Purpose:**

To assess surgically induced astigmatism (SIA) after XEN gel stent implantation over 5 visits during a 3-month follow-up. Changes in intraocular pressure (IOP), IOP-lowering medications, and best-corrected visual acuity (BCVA) were also assessed.

**Methods:**

This prospective cohort study recruited 24 eyes from 24 glaucoma patients at KKU Eye Center, Khon Kaen University, Thailand. All eyes underwent XEN implantation, using our specific surgical technique. We evaluated both the magnitude and the axis of SIA at 1, 2 weeks, 1, 2 and 3 months after the procedure. Preoperative and postoperative intraocular pressure (IOP), IOP-lowering medications, and best-corrected visual acuity (BCVA) were also analyzed.

**Results:**

We observed a statistically significant centroid SIA of 0.14 D. at an axis of 105° and median SIA of 0.36 D. at 1 week following XEN (p < 0.01). However, no further significant statistical change in SIA was found at 2 weeks, and 1, 2, and 3 months postoperatively. Additionally, there was no significant change in BCVA after XEN. Moreover, reductions in IOP and IOP-lowering medications were statistically significant.

**Conclusions:**

Ab Interno XEN gel stent implantation induces a small SIA immediately after the surgery, but no further significant change during 3-month follow-up period. Although SIA has no significant effect on visual acuity, this should be addressed with patients preoperatively. Further studies are needed to investigate how different surgical techniques may affect refractive changes after XEN.

## Introduction

Glaucoma is a multifactorial, progressive optic neuropathy and the second leading cause of blindness worldwide, after cataracts. Primary open-angle glaucoma (POAG) affects an estimated 57.5 million people globally [1]. Intraocular pressure (IOP) is the only known modifiable risk factor for glaucoma, while other significant risk factors include older age, nonwhite race, and a family history of the disease [2]. Effective glaucoma management primarily focuses on lowering IOP, which can be achieved through medication, laser treatment, or surgery [3]. Traditional surgical treatments, such as trabeculectomy with mitomycin C and glaucoma drainage devices, remain the standard options [4].

Minimally invasive glaucoma surgery has introduced innovative procedures designed to reduce IOP with a safer profile compared to conventional surgical methods. One such procedure is the implantation of the XEN gel stent, which has shown effectiveness and safety in lowering IOP and reducing the need for IOP-lowering medications [5–7]. The XEN stent is typically inserted using an *ab interno* approach, involving a small corneal incision. This incision, along with any associated scleral or conjunctival manipulations, can alter the corneal shape and potentially lead to surgically induced astigmatism (SIA).

Although previous studies have demonstrated the effectiveness of XEN implantation [5–7], research on astigmatic changes following the surgery is limited and inconclusive. Tatti et al. reported no significant changes in keratometry readings during a three-month follow-up period after XEN implantation [8], whereas Bormann et al. observed significant SIA changes at three months post-surgery [9]. Generally, astigmatism develops immediately after surgery and stabilizes within 4–12 weeks. Therefore, the three-month follow-up in Bormann’s study may have been too late to capture immediate SIA changes following XEN implantation.

To address this gap, we aimed to investigate SIA changes at earlier time points, focusing on both the magnitude and direction of these changes and the time required for stabilization, using our specific surgical techniques for XEN implantation.

## Materials and methods

This prospective study was reviewed and approved by the Khon Kaen University Ethics Committee for Human Research (Ethics approval code: HE631089), with all procedures adhering to the Declaration of Helsinki. The study recruited glaucoma patients who underwent XEN implantation at KKU Eye Center, Khon Kaen University, Thailand, from July 10, 2020, to October 1, 2023. All patients provided signed written informed consent to participate in the study. Inclusion criteria were: patients with open angle glaucoma and primary angle closure glaucoma with pseudophakic status, aged over 18 years, selected for XEN implantation, and no prior ocular surgeries other than uncomplicated phacoemulsification cataract surgery. Exclusion criteria included congenital glaucoma, prior ocular surgeries other than phacoemulsification, corneal diseases, and intraocular neovascularization. Withdrawal criteria were ocular or head trauma post-XEN implantation and complications such as stent displacement, stent tear, or choroidal detachment.

We used G*Power software to calculate the sample size required to detect clinically meaningful changes in surgically induced astigmatism (SIA) over five follow-up visits within three months after XEN implantation. Based on prior research [10], we estimated the average meaningful SIA to be 0.5 diopters. With a two-sided significance level of 0.05 and 80% power, the sample size was calculated to be at least 21 eyes needed for the study. To account for possible dropouts, we added a 15% buffer and recruited 24 eyes in total.

The decision to perform XEN implantation was made collaboratively between the patient and surgeon, based on the patient’s disease status and the most suitable treatment option. Patients were thoroughly informed about the procedure, including its risks and benefits. Recruitment occurred after XEN procedures were scheduled, ensuring patient participation was independent and free from conflicts of interest.

### Surgical procedures

The XEN microstent was implanted through a temporal clear corneal incision 1–2 millimeters anterior to the limbus, created using a 15-degree blade, with a paracentesis wound length of 1.8 mm. Only one eye was surgically treated in each patient by the same surgeon (PS). For the right eye, the paracentesis site was at the 9 o’clock position, and for the left eye, it was at 3 o’clock. Viscoelastic agents were injected to stabilize the anterior chamber depth. The XEN implant (Allergan, Irvine, CA, USA) was inserted into the anterior chamber and advanced toward the 12 o’clock position of the trabecular meshwork until it was visible in the superonasal quadrant of the subconjunctival space. Viscoelastic agents were then removed and replaced with balanced salt solution.

### Data collection

Data were collected preoperatively and at 1 and 2 weeks, as well as at 1, 2, and 3 months postoperatively. Collected variables included age, gender, glaucoma type, IOP, and the number of IOP-lowering medications. Best-corrected visual acuity (BCVA) was recorded in Snellen notation and converted to logarithm of the minimum angle of resolution (logMAR) for analysis. Objective refraction was reported as spherical equivalent, calculated by adding the sphere power with half of the cylinder power, in diopter (D) unit.

### Assessment of surgically induced astigmatism

During 5 visits in the study, we measured both the magnitude and the axis of corneal astigmatism using an auto Ref/Keratometer (Nidek, Aichi, Japan). Keratometry values were repeated and averaged. Data on individual astigmatism were recorded on an Excel spreadsheet (Corneal SIA Tool V.1.0.0 spreadsheet) provided by the American Society of Cataract and Refractive Surgery (ASCRS), based on the method outlined by Koch et al. [11,12]. The worksheet automatically generated double-angle plots of the corneal SIA values. Centroid values, standard deviations, and 95% confidence ellipses of the centroid and the dataset were calculated. Mean absolute errors with standard deviations of the data were also displayed.

### Statistical analysis

Statistical analysis was performed using R (version 4.3.0) and R Studio. For numerical data, the Shapiro-Wilk test was used to check for normality of the data. Age was presented as mean and standard deviation, while IOP, the number of IOP-lowering medications, BCVA, spherical equivalent and SIA were presented as median and interquartile range. Due to non-normal distribution of the data, Friedman’s test was used to assess differences between time points, while the Nemenyi test was applied for pairwise comparisons during the postoperative course. SIA was specifically analyzed in both median and centroid values. Median SIA was based on only the magnitude of astigmatism, whereas centroid value was based on both the magnitude and the axis of astigmatism. A p-value of less than 0.05 was considered statistically significant.

## Results

Out of 28 patients who consented to the study, four patients did not have complete postoperative refractive status data and were excluded from the analysis. Among the 24 patients, most patients had primary and secondary open angle glaucoma. There were two patients with primary angle closure glaucoma and both of them were pseudophakic at the time of XEN gel stent implantation. Other baseline characteristics of patients are presented in Table 1.

**Table 1:** Baseline characteristics of patients.

Baseline characteristics	n = 24
Age (years) *Mean (SD)*	56.4 (15.0)
Gender *N (%)*	
Female	5 (20.8)
Male	19 (79.2)
Types of Glaucoma *N (%)*	
Primary open angle glaucoma	15 (62.5)
Juvenile open angle glaucoma	4 (16.7)
Secondary open angle glaucoma	1 (4.2)
Acute angle closure glaucoma	1 (4.2)
Angle recession glaucoma	1 (4.2)
Chronic angle closure glaucoma	1 (4.2)
Uveitis-glaucoma-hyphema	1 (4.2)
Lens status *N (%)*	
Phakic	16 (66.7%)
Pseudophakic	8 (33.3%)
Intraocular Pressure (mmHg) *Median (IQR)*	18 (16, 25)
Best corrected visual acuity (logMAR) *Median (IQR)*	0.35 (0, 0.53)
Sphere (diopters) *Median (IQR)*	−0.75 (−3.5, 0.56)
Astigmatism (diopters) *Median (IQR)*	−0.88 (−1.56, −0.69)

Abbreviation: SD = standard deviation, logMAR = logarithm of the minimum angle of resolution, IQR = interquartile range.

### Surgically induced astigmatism (SIA)

**Fig 1:**
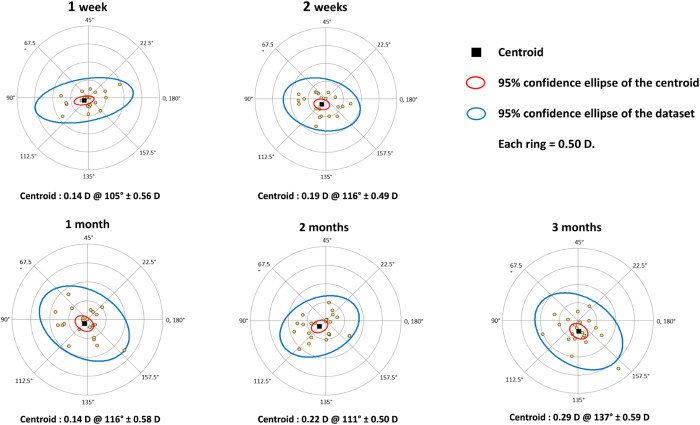
Double angle plots showing individual surgically-induced astigmatism vectors at 1, 2, and 3 months after surgery. Each dot represents one patient’s SIA. The centroid (average) vector is shown for each time point.

**Fig 2:**
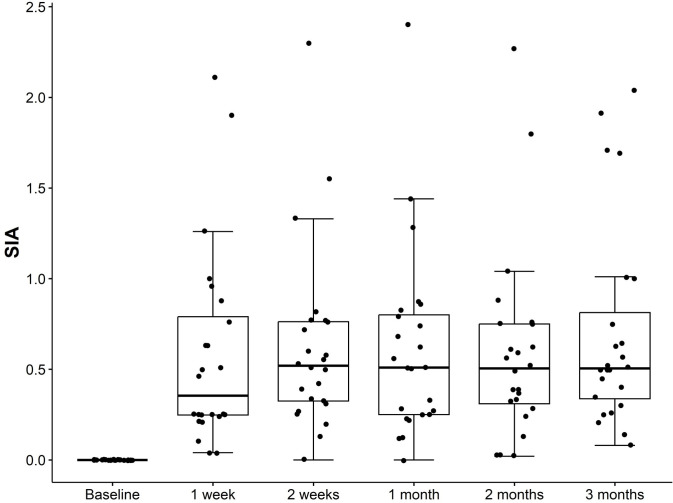
Box and whiskers plot of the magnitude of surgically-induced astigmatism at 1, 2 weeks, 1, 2, and 3 months after surgery. The box represents the IQR, the median is marked as a line within the box, and whiskers extend to the highest and lowest scores within the 1.5 IQR range. Dots outside the box and whiskers represent outliers.

We analyzed 24 eyes from 24 patients who underwent XEN implantation. Data from all 5 visits within the first 3-month postoperative follow-up were obtained. The double angle plots and centroid values of the patients at 1, 2 weeks, and at 1, 2, and 3 months after surgery are shown in Fig 1. The centroid value represents the central tendency of the SIA vectors, providing both the average amount and direction of the SIA. In this study, centroid value of the SIA was 0.14 ± 0.56 diopter (D) at an axis of 105° at 1 week. However, the value of 0.29 ± 0.59 D at an axis of 137° at 3 months after surgery was not statistically significant as compared to the value at 1 week. Similarly, significant median SIA of 0.36 (0.25, 0.79) D (p < 0.01) was observed at 1 week postoperatively. However, at 2 weeks, and at 1, 2, and 3 months postoperatively, no significant change in existing SIA was found as compared to those at 1 week (Table 2 and Fig 2).

**Table 2:** Surgically induced astigmatism after XEN gel implantation. After Friedman test shows that SIA are significantly different between time points (p < 0.01), Nemenyi test is used for pairwise comparison to identify that SIA at 1 week is different from baseline (p < 0.01).

	SIA (Diopter)	p-value of Friedman’s test	Nemenyi test
Baseline	0	<0.01	Ref
1 week	0.36 (0.25, 0.79)		<0.01
2 weeks	0.52 (0.33, 0.76)		0.98
1 month	0.51 (0.25, 0.80)		0.99
2 months	0.51 (0.31, 0.75)		0.99
3 months	0.51 (0.34, 0.81)		0.78

Abbreviation: SIA = surgically induced astigmatism.

### Spherical equivalent

The median spherical equivalent was −0.75 (−3.5, 0.56) D at baseline, and −0.75 (−3.81, 0.81) and −0.75 (−3.5, 0.81) D at 1 and 3 months, respectively. The change in median spherical equivalent was not statistically significant in all five visits during the first 3-month postoperative follow-up.

### Best-corrected visual acuity

**Fig 3:**
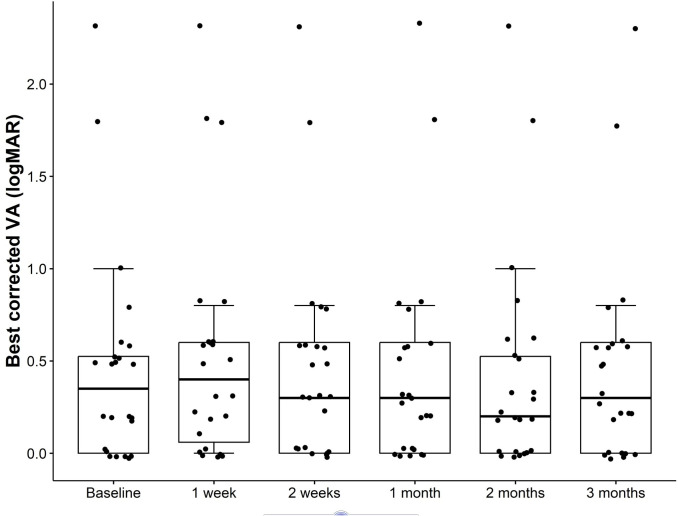
Box and whiskers plot of the preoperative and postoperative BCVA. The box represents the IQR, the median is marked as a line within the box, and whiskers extend to the highest and lowest scores within the 1.5 IQR range. Dots outside the box and whiskers represent outliers.

There were no significant changes in best-corrected visual acuity (BCVA) postoperatively. The median logMAR BCVA was 0.35 (0, 0.53) preoperatively and 0.3 (0, 0.53) at the last follow-up (p = 0.83) (Fig 3).

### IOP and IOP-lowering medications

**Fig 4:**
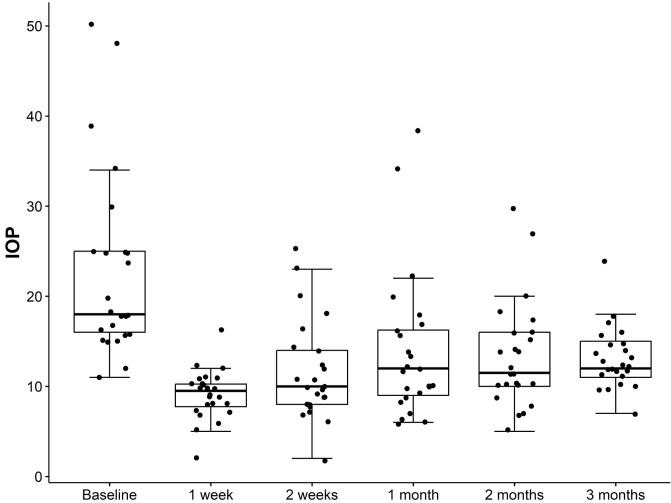
Box and whiskers plot of the preoperative and postoperative IOP. The box represents the IQR, the median is marked as a line within the box, and whiskers extend to the highest and lowest scores within the 1.5 IQR range. Dots outside the box and whiskers represent outliers.

**Fig 5:**
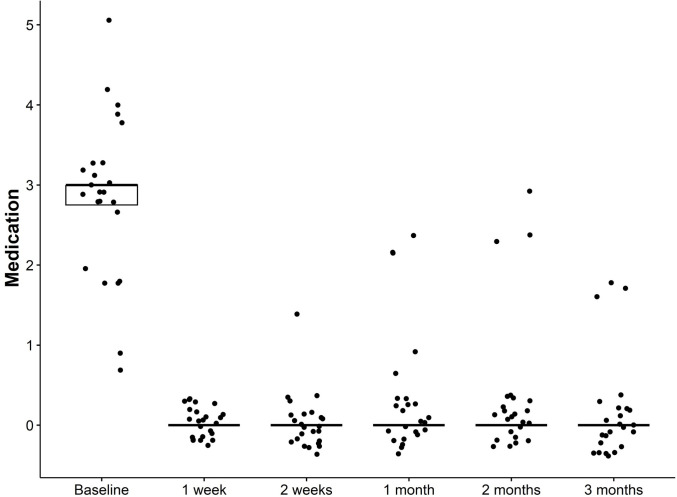
Box and whiskers plot of the preoperative and postoperative IOP-lowering medication. The box represents the IQR, the median is marked as a line within the box, and whiskers extend to the highest and lowest scores within the 1.5 IQR range. Dots outside the box and whiskers represent outliers.

Median baseline IOP was 18 (16, 25) mmHg, decreasing to 12 (11, 15) mmHg at 3 months after XEN implantation, a significant reduction (p < 0.001) (Fig 4). IOP-lowering medications decreased from 3 (2.75, 3) preoperatively to 0 (0, 0) at the last follow-up (p < 0.001) (Fig 5).

## Discussion

Our prospective study evaluated the effect of XEN gel stent implantation on surgically induced astigmatism (SIA) in 24 eyes. It demonstrated a centroid value of 0.14 D at an axis of 105° and a significant median SIA of 0.36 D as early as one week postoperatively, with no significant differences in SIA compared to baseline during subsequent follow-up visits. Interestingly, it was noted that centroid value was markedly smaller than median SIA in all visits. This was attributable to the double angle plots of individual SIA which showed various astigmatic vectors in magnitude and direction, and thus resulted in counteraction. This reduction was in accordance with previous study [13]. Kamiya et al. compared mean and centroid value of SIA after standard cataract surgery and demonstrated that centroid value reduced to approximately 40% of the mean SIA [13].

This research provides insights into the short-term refractive outcomes following XEN implantation, an area that has been limited in the current literature. Tatti et al. studied keratometry changes after XEN implantation in 20 patients with primary open-angle glaucoma over a 3-month follow-up period and found no significant changes in total astigmatism [8]. However, Bormann et al., in a retrospective study, reported significant SIA changes of 0.79 D at 3 months [9]. The purpose of Bormann’s study was to compare visual acuity and refractive changes after trabeculectomy or XEN implantation over a 24-month follow-up period. Their results showed significant SIA in both groups at 3 months postoperatively, with no further significant changes in SIA at 6, 12, and 24 months [9].

This discrepancy in SIA outcomes may be attributed to differences in surgical techniques. Tatti et al. inserted the injector needle through a 1.8-mm inferotemporal corneal incision, directing it toward the superonasal quadrant for XEN implantation [8]. In contrast, Bormann et al. used two 1.1-mm paracenteses: one located in the inferotemporal quadrant and the other nasally, stabilizing the eye during XEN implantation in the superonasal quadrant [9]. Our study employed a 1.8-mm paracentesis at the 3 or 9 o’clock position, with the XEN stent exiting at the 12 o’clock position of the trabecular meshwork. These variations likely contribute to differing SIA outcomes.

Our surgical technique utilized a temporal approach, similar to the temporal clear corneal incisions used in phacoemulsification, known to induce minimal postoperative astigmatism [14–17]. Dick et al. conducted a prospective, randomized study in 60 patients, comparing SIA after 3.5-mm, 4.0-mm, and 5.0-mm temporal corneal incisions over six months [14]. They found that temporal corneal incision resulted in minimal astigmatism and smaller incisions had significantly lower SIA [14]. Similarly, Pfleger et al. studied postoperative astigmatism in 103 cases and reported that the smallest incision (3.2 mm) induced the least SIA [15]. Masket et al. conducted a prospective, randomized controlled study in 22 patients who underwent clear corneal cataract surgery in both eyes. One eye received a 2.2-mm incision, while the fellow eye received a 3.0-mm incision. Their findings showed a statistically and clinically significant reduction in SIA with the smaller incision size [16]. Furthermore, Yao et al. compared microincisions (1.7 mm) to small incisions (3.2 mm) and found that microincisions resulted in less corneal astigmatism and better optical quality [17]. Jin et al. also concluded that microincisions might induce slightly less astigmatism than standard incisions, but the difference (approximately 0.2 D) did not require correction [18,19].

The lower median SIA observed in our study may be due to the use of a temporal microincision. Besides surgical technique, preoperative corneal curvature may influence SIA. Jiang et al. demonstrated that corneal topography-guided clear corneal incision phacoemulsification can yield better visual outcomes by reducing pre-existing astigmatism and inducing less postoperative astigmatism than conventional temporal incisions [20]. Additionally, initial SIA changes may result from early hypotony, bleb-related effects, postoperative inflammation, changes at the scleral-corneal junction, and early healing responses. Monitoring these refractive changes is essential for optimizing postoperative visual outcomes.

In general, small corneal incisions do not alter spherical power but can induce significant astigmatism and trefoil aberration [21]. However, these changes typically stabilize or partially revert to preoperative values by the third month postoperatively [21]. Compared to trabeculectomy, the degree of SIA after XEN implantation is generally smaller, stabilizing within a few months [9]. Our study identified small SIA, both median SIA and centroid value, at one week post-XEN implantation, with no further significant changes during subsequent follow-ups, suggesting early stabilization of refractive changes. This finding supports the XEN stent as a safer option for preserving visual acuity while managing glaucoma.

Additionally, our study showed significant reductions in IOP and the number of IOP-lowering medications used postoperatively, consistent with previous studies on the efficacy of the XEN gel stent in glaucoma management [5–7,9]. The median baseline best-corrected visual acuity (BCVA) remained stable throughout the three-month follow-up period. In comparison, visual acuity was found to be decreased three months post-trabeculectomy, suggesting a potential advantage of XEN over trabeculectomy [9].

The strengths of our study are the prospective study design and investigation of SIA changes at earlier time points so that to capture immediate astigmatic change. We evaluate both the magnitude and the direction of these changes by vector analysis and the astigmatism double angle plot, which provide more accurate SIA. Furthermore, we also present our specific surgical techniques for XEN implantation which induces less SIA change and early stabilization. However, there are some limitations that should be mentioned. Using the auto Ref/Keratometry in this study would not exclude patients with corneal ectasia. Corneal topography should be performed to detect irregularities in the shape and curvature of the cornea. Additionally, our study was limited to a 3-month follow-up period, and the predominantly Asian population may limit generalizability to other ethnic groups. Future research should include extended follow-up periods to evaluate the long-term results and its impact on visual acuity and patient satisfaction. Comparative studies with other minimally invasive glaucoma surgeries could help determine the relative benefits and drawbacks of different procedures. Investigations into alternative surgical techniques may identify methods to further minimize postoperative SIA. Additionally, studies across diverse populations are needed to assess the influence of demographic factors such as ethnicity, age, and glaucoma type on surgical outcomes. Addressing these areas will help refine clinical practices and improve patient outcomes in glaucoma management.

## Conclusion

In conclusion, XEN gel stent implantation is a safe and effective procedure for lowering IOP in glaucoma patients. Our surgical technique induces a small SIA immediately after the surgery, which returns to non-significant levels after the first week and has no significant effect on visual acuity. Although small SIA is not meaningful for patients, these findings are crucial for preoperative patient counseling and surgical planning, ensuring that patients have realistic expectations regarding the refractive outcomes of the procedure.

## Supporting information

S1 File Dataset. (XLSX)

S2 File STROBE checklist cohort. (DOCX)
